# The Therapeutical Estimate Determined upon Cord. 01. Morrhuae Comp. (Hagee)

**Published:** 1897-08

**Authors:** Willard H. Morse

**Affiliations:** Iamatological Chemist, Westfield, N. J.


### Therapeutic Notes.

---

### The Therapeutical Estimate Determined upon Cord. Ol. Morrhuae Comp. (Hagee).

---

BY WILLARD H. MORSE, M. D., F. S. S., IAMATOLOGICAL
CHEMIST, WESTFIELD, N. J.

---

I. A careful analysis shows the presence therein of those definite alkaloids and active medicinal principles obtainable from fresh cod liver oil (and to which such oil owes its medicinal qualities), in combination with the hypophosphites of lime and soda. Therefore,

II. The cordial possesses the therapeutical characteristics of these components, with such physiological actions as are agreeable thereto.

III. By the same analysis it is shown that in the process of manufacture all and every impure and deleterious element has been removed. Therefore,

IV. The cordial produces none but the most positive results, and can be administered to any patient, and indefinitely without creating any repugnance to its use.

V. It is indicated in all forms of wasting diseases and asthenic conditions.

VI. It stimulates and supports assimilative nutrition.

VII. It exerts its influence of an antiseptic and germicide on all micro-organisms.

VIII. It has an effect at once marked, immediate, progressive and continuous.

IX. It obviates all degenerative changes.

X. An agreeable preparation, readily taken, and fully serviceable, it is to be appreciated as a very important addition to the new materia medica.

